# Supplementary material for: Crystallization of piezoceramic films on glass via flash lamp annealing
Source: Nat Commun. 2024 Feb 29;15:1890. doi: 10.1038/s41467-024-46257-0 (PMC10904753; doi:10.1038/s41467-024-46257-0)
Supplement: Supplementary file 1 — Supplementary Information [file 41467_2024_46257_MOESM1_ESM.pdf]

Supplementary Information for

Crystallization of piezoceramic films on glass via flash lamp annealing

Longfei Song,[1,2,†] Juliette Cardoletti,[1,†] Alfredo Blázquez Martínez,[1,2] Andreja Benčan,[3] Brigita Kmet,[3] Stéphanie Girod,[1] Emmanuel Defay,[1] Sebastjan Glinšek[1,*]

[1] Materials Research and Technology Department, Luxembourg Institute of Science and Technology, 41 rue du Brill, L-4422 Belvaux, Luxembourg

[2] University of Luxembourg, 41 rue du Brill, L-4422 Belvaux, Luxembourg

[3] Electronic Ceramics Department, Jožef Stefan Institute, Jamova cesta 39, 1000 Ljubljana, Slovenia

[†] These authors contributed equally: L. S. and J. C.

*Corresponding author: sebastjan.glinsek@list.lu

Contents

Supplementary Note 1: Finite element modelling (FEM) 3

Supplementary Note 2: Films grown on fused silica glass 4

Supplementary Note 2.1: Phase composition and microstructural characterizations ..4

Supplementary Note 2.2: Electrical measurements..8

Supplementary Note 2.2.1: 170 nm-thick film grown on fused silica glass.......................8

Supplementary Note 2.2.2: 500 nm-thick film grown on fused silica glass.....................10

Supplementary Note 2.3: Comparison of properties at different thicknesses.............11

Supplementary Note 3: Thick PZT film on AF32 glass for surface haptic device...........11

Supplementary Note 3.1: Phase composition and microstructural characterization..11

Supplementary Note 3.2: Haptic devices 13

Supplementary Note 3.2.1: Thickness of piezoelectric film..13

Supplementary Note 3.2.2: Finite element modelling...13

Supplementary Note 3.2.3: Device performance 14

Supplementary Note 3.3: Electrical measurements...16

Supplementary Note 4: Flash lamp process for growing films on soda lime glass 17

Supplementary Note 4.1: Two-step flash lamp annealing process 17

Supplementary Note 4.2: Phase composition characterization 17

Supplementary Note 4.3: Electrical measurements...18

Supplementary Note 5: Comparison with previous works 19

Supplementary Note 1: Finite element modelling (FEM)

In order to investigate the feasibility of crystallization of $PbZr_{0.53}Ti_{0.47}O_3$ (PZT) thin films via flash lamp annealing, finite element modelling was performed using surface mode of SimPulse software to study the temperature evolution across the thickness of the samples[1].

To estimate the absorbance inside amorphous PZT films, a method using a bolometer was utilized[2]. While this method is not ideal, it is a reasonable approach as the measurement is performed under the same conditions as flash lamp annealing. First, the energy density on the surface of the stage of the flash-lamp annealer was measured with a bolometer after the light passed through air (direct exposure to light, E_{tot} = 2.8 J cm^{-2}), bare fused silica glass (estimation of light reflected from the glass substrate assuming negligible absorption, $E_{glass} = E_{tot} - E_{ref}$ = 2.6 J cm^{-2}), and a fused silica glass with amorphous PZT on top (estimation of light that passes through the stack, $E_{sample} = E_{trans}$ = 1.8 J cm^{-2}), see Supplementary Figure 1. To estimate the quantity of absorbed light, we used the equation:

$$E_{\mathrm{abs}} = E_{\mathrm{tot}} - E_{\mathrm{sample}} - E_{\mathrm{ref}} = E_{\mathrm{tot}} - E_{\mathrm{trans}} - (E_{\mathrm{tot}} - E_{\mathrm{glass}}). \quad (1)$$

In equation (1), E denotes energy density in J cm^{-2}. The resulting absorbed energy E_{abs} is 0.8 J cm^{-2}. The corresponding absorption value A is estimated as:

$$A = \frac{E_{\mathrm{abs}}}{E_{\mathrm{tot}}}, \quad (2)$$

which was calculated as 28.5 %. Note that this value is a rough estimation only.

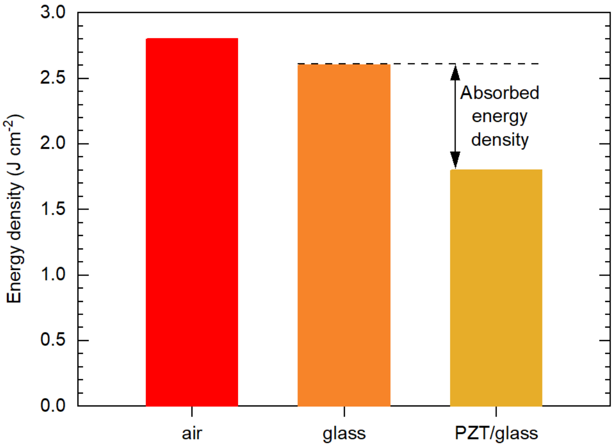

Supplementary Figure 1. **Transmitted energy density** measured with a bolometer after the light passing through: air (empty chamber), 500 µm-thick fused silica glass, and pyrolyzed PZT/fused silica stack. The energy density and the length of the applied light pulse were 2.8 J cm^{-2} and 130 µs.

Supplementary Note 2: Films grown on fused silica glass

Supplementary Note 2.1: Phase composition and microstructural characterizations

A standard θ-2θ X-ray diffraction (XRD) pattern of a flash lamp annealed 1 µm-thick PZT film on fused silica is shown in Supplementary Figure 2. Presence of piezoelectrically active perovskite phase is revealed, and all the reflections can be identified with the PZT powder diffraction file (PDF) No 01-070-4264[3]. Reflections of secondary phases are not observed.

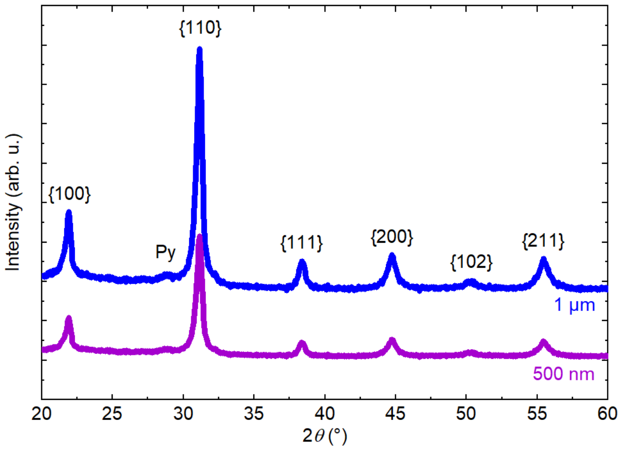

Supplementary Figure 2. **XRD study**. θ-2θ XRD patterns of flash lamp annealed 500 nm and 1 μm PZT films on fused silica glass. The films were processed with 50 pulses per layer (3 and 6 crystallizations in total, respectively). Parameters of each pulse are reported in caption of Fig. 2 in the main manuscript. The PDFs No 01-070-4264 and 04-014-5162[3] have been used to identify the perovskite and pyrochlore phases, respectively.

Grazing incidence X-ray diffraction (GIXRD) pattern of the film annealed in a conventional RTA furnace at 700 °C is shown in Supplementary Figure 3. Only pyrochlore phase is detected and cracks appeared on the surface, as previously reported in Ref.[4].

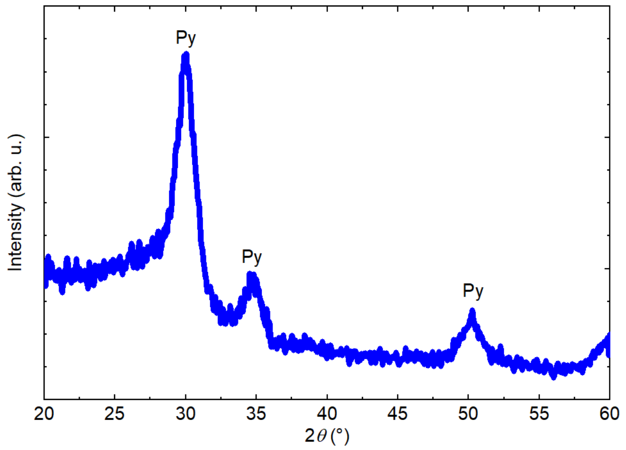

Supplementary Figure 3. **XRD study**. GIXRD pattern of a conventionally RTA-processed film on fused silica at 700 °C. Py indicates the pyrochlore reflection according to PDF No 04-014-5162[3].

The transmittance spectra of PZT thin films of various thicknesses on fused silica glass are shown in Supplementary Figure 4. The inset shows that 1 µm-thick PZT films are transparent.

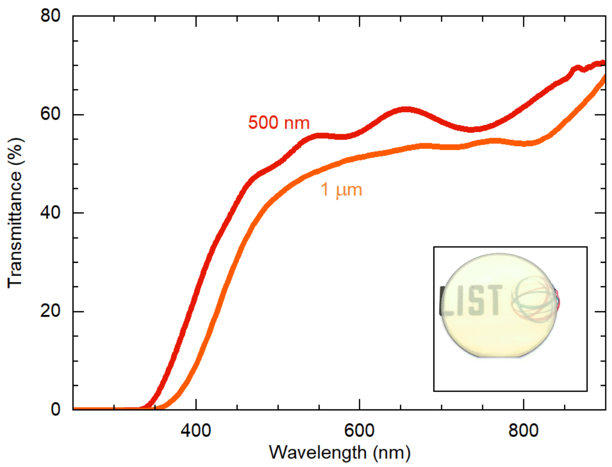

Supplementary Figure 4. **Transmittance of 500 nm and 1 µm-thick PZT thin films on fused silica glass.** Inset shows the optical appearance of the 1 µm-thick film. The films were processed with 50 pulses per layer (3 and 6 crystallizations in total, respectively). Parameters of each pulse are reported in caption of Fig. 2 in the main manuscript.

Supplementary Figure 5 displays cross-sectional scanning electron microscopy (SEM) image of a flash lamp annealed 1 µm-thick PZT, showing a clean interface between PZT film and glass. The microstructure is granular with present porosity.

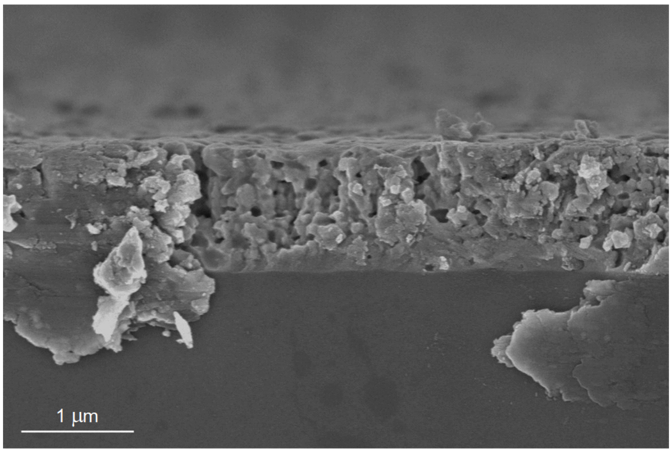

Supplementary Figure 5. **Microstructure characterization**. Cross-sectional SEM image of flash lamp annealed 1 µm PZT film on fused silica glass. Note that the large flake-like structures observed in the glass are due to glass cleaving.

A detailed TEM analysis of the 170 nm-thick PZT thin film on fused silica glass is shown in Supplementary Figure 6.

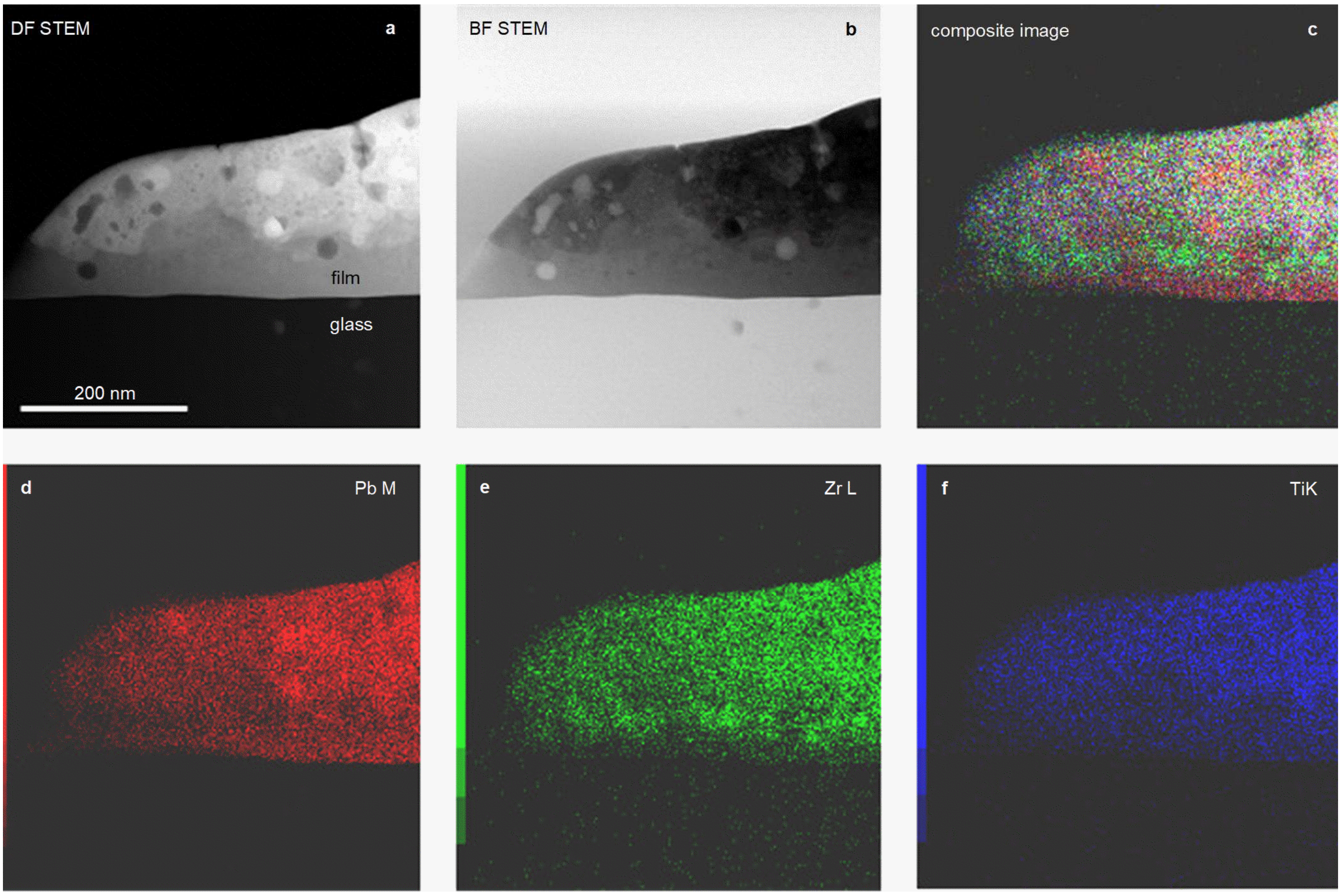

Supplementary Figure 6. **TEM analysis of 170 nm PZT thin film on fused silica glass**. a) Cross sectional dark-field (DF) and b) bright-field (BF) scanning transmission electron microscope (STEM) image with c-f) corresponding energy-dispersive X-ray spectroscopy system mapping showing porous, chemically non-homogenous film. Pores are darker/brighter spots on DF/BF STEM images, correspondingly.

Supplementary Note 2.2: Electrical measurements

Supplementary Note 2.2.1: 170 nm-thick film grown on fused silica glass

We found that the films annealed with 50 pulses exhibit the optimal electrical properties. Polarization as a function of electric field $P(\mathbf{E})$ and corresponding current density $J(\mathbf{E})$ loops of the 170 nm-thick films treated with 50 pulses at 100 and 10 Hz are shown in Supplementary Figure 7. The polarization loops of the films are initially pinched, before opening up during

(wake-up effect) upon electric-field cycling (1.1×10^6 bipolar cycles). At 10 Hz, the P_r is 10 µC cm^{-2}, and two sharp peaks in J(**E**), linked to ferroelectric switching, are observed.

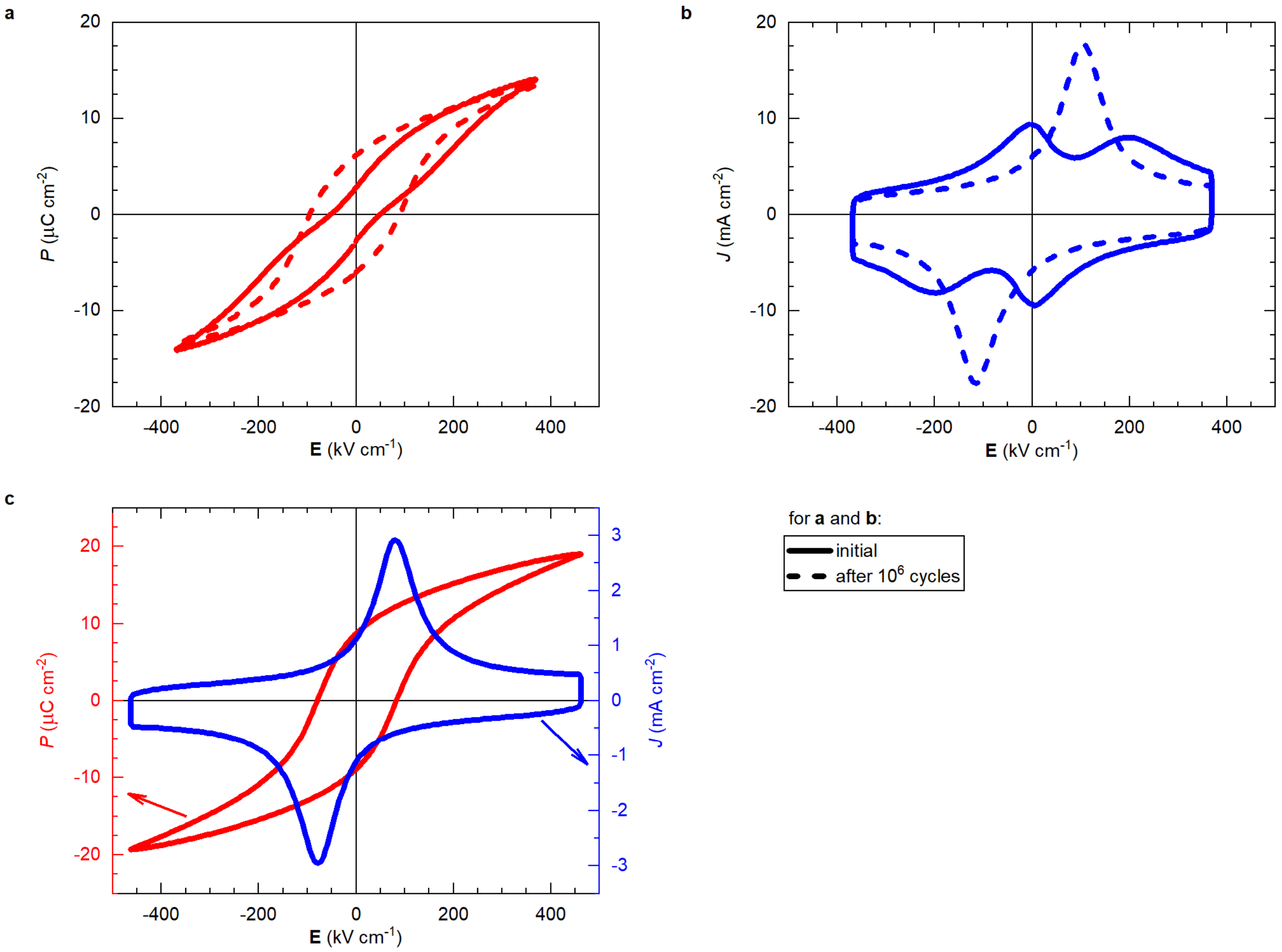

Supplementary Figure 7. **Ferroelectric characterization**. a) Polarization P(**E**) hysteresis loops of the 50 pulses annealed 170 nm-thick PZT film on fused silica before and after 1.1×10^6 wake-up cycles, measured at 100 Hz. b) corresponding current density loops J(**E**). c) P(**E**) and J(**E**) measured at 10 Hz, by taking the average after 500 cycles. Large interdigitated electrodes (IDE) were used in these measurements, corresponding to an effective area of 0.36 mm^2.

Supplementary Note 2.2.2: 500 nm-thick film grown on fused silica glass

The P(**E**) and J(**E**) loops of the 500 nm-thick PZT film on fused silica glass are shown in Supplementary Figure 8a with a maximum polarization P_{max} of 21 μC cm^{-2} and a remanent polarization P_r of 11 μC cm^{-2}. Its coercive field E_c is 95 kV cm^{-1}. Note that these values were obtained after 10^3 wake-up cycles. The displacement of a cantilever structure shows a typical butterfly loop (Supplementary Figure 8b). At 150 V the vertical displacement at the free end of the cantilever is 625 nm, corresponding to a piezoelectric coefficient $e_{33,f}$ of -5 C m^{-2} [5].

All the results confirm good ferroelectric properties of 50 pulses flash lamp annealed films.

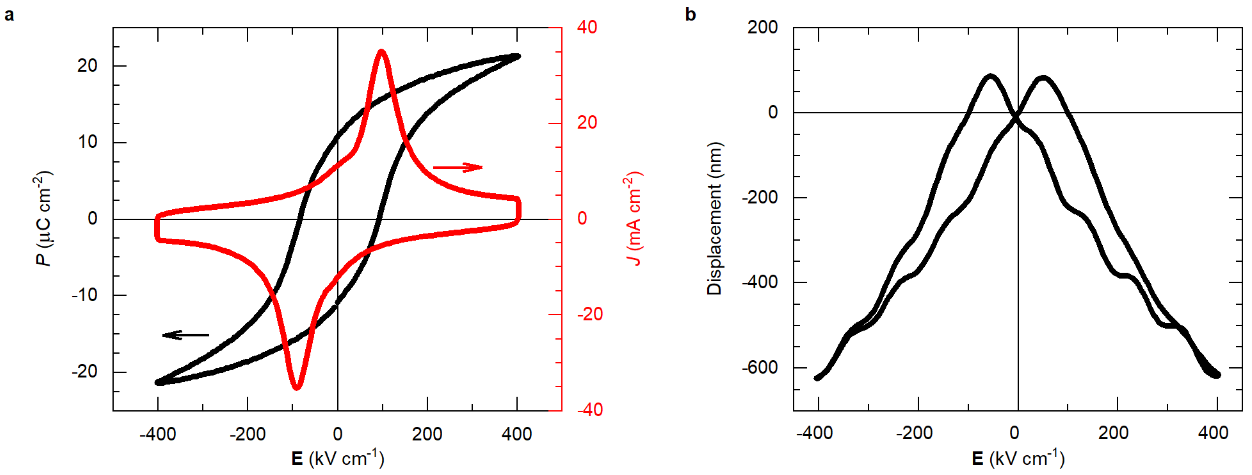

Supplementary Figure 8. **Electromechanical characterization of a 500 nm-thick PZT film on fused silica glass**. a) Ferroelectric and b) displacement characterizations of the PZT film at 100 Hz and 11 Hz, respectively. The samples were processed with 50 pulses per layer with energy density, pulse duration and repetition rate of 3 J cm^2, 130 μs, and 3.5 Hz, respectively.

Supplementary Note 2.3: Comparison of properties at different thicknesses

To have better overview of the results, a table with ferroelectric, piezoelectric and optical properties of the 170 nm, 500 nm and 1 µm-thick films on fused silica substrates is provided in Supplementary Table 1.

Supplementary Table 1. **Properties of 170 nm, 500 nm and 1 µm-thick PZT films on fused silica glass**. Remanent and maximum polarization (P_r and P_{max}) at an applied voltage of 150 V, relative permittivity and dielectric losses (ε_r and tanδ), piezoelectric coefficient $e_{33,f}$, and transmittance (T) at a wavelength of 550 nm. The films were processed with 50 pulses per layer with energy density, pulse duration and repetition rate of 3 J cm^{-2}, 130 µs, and 3.5 Hz, respectively. Large IDEs were used in these measurements, corresponding to an effective area of 0.36 mm^2, 1.08 mm^2, and 2.16 mm^2 for 170 nm, 500 nm and 1 µm, respectively.

Film thickness	P_r (µC cm^{-2})	P_{max} (µC cm^{-2})	ε_r	tanδ	$e_{33,f}$ (C m^{-2})	T (%)
170 nm	10	19	200	0.05	-2	64
500 nm	11	21	270	0.05	-5	56
1 µm	12	24	450	0.05	-5	49

Supplementary Note 3: Thick PZT film on AF32 glass for surface haptic device

Supplementary Note 3.1: Phase composition and microstructural characterization

A 1 µm-thick PZT film was grown on AF32 glass. Process parameters were the same as for the films on fused silica, namely 3 J cm^{-2} in energy density, 130 µs pulse duration and 50 pulses with a repetition rate of 3.5 Hz. Supplementary Figure 9 shows θ-2θ XRD pattern of the film. Reflections of the perovskite phase are revealed and all of them can be identified with the PZT PDF No 01-070-4264[3], as for the film grown on fused silica. Note that the reflection at around 39° comes from the Pt electrodes.

Supplementary Figure 10 shows cross-sectional SEM image of a 1 µm-thick PZT film on AF32 glass, used for haptic device. It reveals a dense and granular microstructure, and also a clear interface between the film and glass.

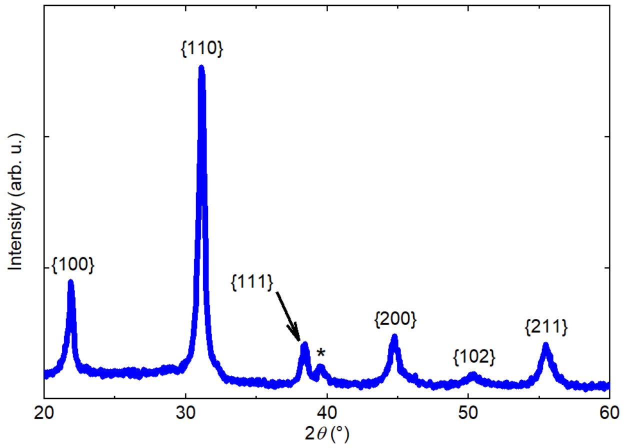

Supplementary Figure 9. **XRD study**. θ-2θ XRD pattern of flash lamp annealed 1 µm thick PZT film on AF32 glass used for the haptic device. * denotes the signal of Pt IDEs on top of the PZT film. The PDFs No 01-070-4264[3] has been used to identify the perovskite phase.

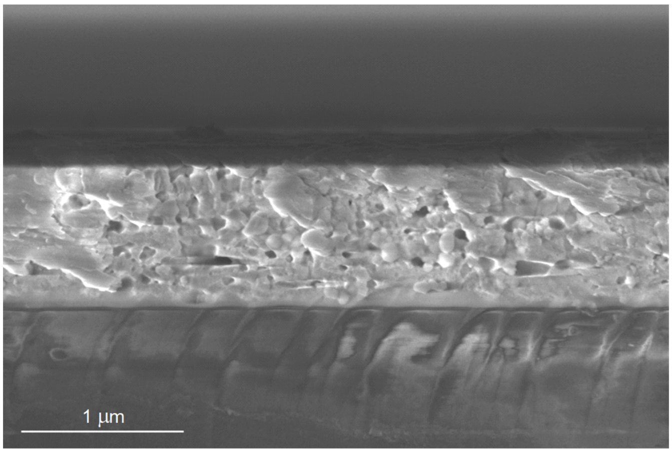

Supplementary Figure 10. **Microstructure characterization**. Cross-sectional SEM image of 1 µm-thick PZT on AF32 glass, used for haptic device. The processing conditions have been described in the Methods.

Supplementary Note 3.2: Haptic devices

Supplementary Note 3.2.1: Thickness of piezoelectric film

In general, piezoelectric films show increased electromechanical response with increasing thickness due to larger contributions of domains[6]. In the case of piezoelectric actuators with interdigitated (IDE) geometry, additional benefit of using thicker film is an increased in-plane force F_3 exerted by the piezoelectric layer upon applied electric field E_3. The force is expressed as:

$$F_3 = -e_{33,\mathrm{f}} E_3 A, \tag{2}$$

where E_3 is an in-plane electric field, $e_{33,\mathrm{f}}$ is an effective piezoelectric coefficient and A is a cross-section. In IDE geometry E_3 roughly equals to an applied voltage U divided by a gap a between the fingers, while A equals to a film thickness t_f multiplied by a finger length l. The above equation can be therefore re-written as:

$$F_3 = -e_{33,\mathrm{f}} \frac{U}{a} l t_\mathrm{f}, \tag{3}$$

from which it follows that the force exerted by IDE piezoelectric actuator (at constant voltage) can be increased by increasing film's thickness (and decreasing the gap between the fingers). Considering these points and ease of processing, we defined 1 μm-thick PZT film as a good compromise.

Supplementary Note 3.2.2: Finite element modelling

Two-dimension (2D) FEM was carried out using COMSOL software to design a haptic device. 1 μm PZT/AF32 glass structure was used in the modelling with the total length of 15.4 mm. Two symmetric actuating areas were created with IDEs with 129 pairs of digits and a spacing of 8.4 mm. The width of the fingers and the interdigital gap are 5 μm and 3 μm, respectively.

Young's modulus and Poisson ratio for AF32 glass are 74.8 GPa and 0.238, respectively[7]. Influence of the electrodes on the deflection were ignored due to their lower thicknesses. The effective transverse piezoelectric coefficient $e_{33,\mathrm{eff}}$ and relative permittivity ε_r were set to

-4.5 C m^{-2} and 400, respectively. Note that $e_{33,\mathrm{eff}}$ was extracted from the modelling by matching the experimental displacement value of the haptic device, after having measured the damping loss factor η[8], which was obtained by sweeping the frequency of the actuator and collecting the displacement, as shown in Fig. 4b. η corresponds to the breadth of the resonance peak ($\Delta f/f$), where Δf is the full-width-at-half-maximum and f is the resonance frequency. The obtained value of η is 0.0163.

At a simulated resonant frequency of 40.2 kHz, the device exhibits a maximum displacement (1.7 µm peak-to-peak) when driven with 60 V. Supplementary Figure 11 illustrates the device's mode shape at resonance, specifically a Lamb wave mode featuring four equally spaced nodes along its length.

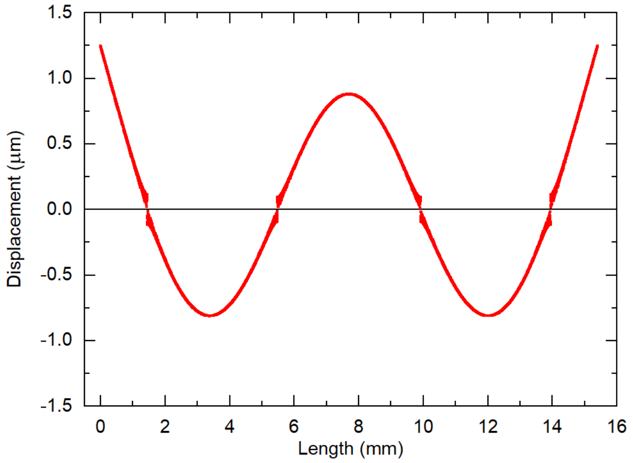

Supplementary Figure 11. **Finite element modelling of haptic device**. Wave shape along the length of the device at resonant frequency.

Supplementary Note 3.2.3: Device performance

The device is operating in a resonance mode and the standing wave correspond to the anti-symmetric (A_0) Lamb wave, which has been considered as optimal for piezoelectric haptics in

previous works[9]. Lamb waves in glass plates for haptics were extensively studied by Bernard[10]. Figure II.3 on page 47 from his work[10] shows wavevectors of Lamb waves as functions of a frequency-thickness product. In our work the glass is 300 μm-thick and is operated at a frequency of 40.2 kHz, leading to the frequency-thickness product of 0.012 MHz mm. This is below the appearance of any other modes than A_0. The fact that Bernard used EAGLE XG and we are using AF32 glass does not change the outcome of this analysis as both glasses have comparable mechanical properties and density.

Performance of the device is compared to other piezoelectric haptic devices on glass with interdigitated geometry in Supplementary Table 2. The three devices are similar in geometry (~3 mm x 15 mm) and have interdigitated electrode structure, which makes the comparison of device performance straightforward. Several points stem from the Table: 1) The device demonstrated in this work operates at lower frequency. This is mainly due to thinner substrate (300 μm vs. 500 μm). 2) Device in this work needs lower U_{rms} to achieve 1 μm displacement. This is mainly due to decreased gap between the fingers (3 μm vs. 10 μm, see Equation (2)) and lower substrate thickness. 3) Higher total capacitance in the current device is mainly due to a combination of smaller gap and finger width (5 μm vs. 10 μm). Most importantly, this device shows similar power consumption (35 mW) compared to the other two devices, which confirms its high quality.

Supplementary Table 2: **Comparison of piezoelectric thin-film haptic devices on glass with interdigitated geometry.** f – resonant (operating) frequen□y; U_{rms} – root mean square (rms) voltage at 1 µm defle□tion; C_{device} – □apa□itan□e of the devi□e; P_{cons} – power consumption estimated as $P_{cons} = C f (U_{rms})^2$. In all three cases thickness of PZT was 1 µm and devices had similar geometries.

Device	Glass	f (kHz)	U_{rms} (V)	C_{device} (pF)	P_{cons} (mW)
Spin-coated[4]	Fused silica	73.0	43	240	32
Inkjet-printed[11]	Fused silica	63.3	46	230	31
This work	AF32	40.2	34	760	35

Supplementary Note 3.3: Electrical measurements

Supplementary Figure 12 shows initial $P(\mathbf{E})$ and $J(\mathbf{E})$ loops for a single haptic actuator. Similarly pinched loops are observed as on fused silica (see Supplementary Figure 7). Note that this initially pinched behaviour does not influence haptic performance, as was demonstrated in inkjet-printed (RTA-processed) PZT films with similar behaviour[12]. Permittivity and losses values at zero field are 350 and 0.07, respectively.

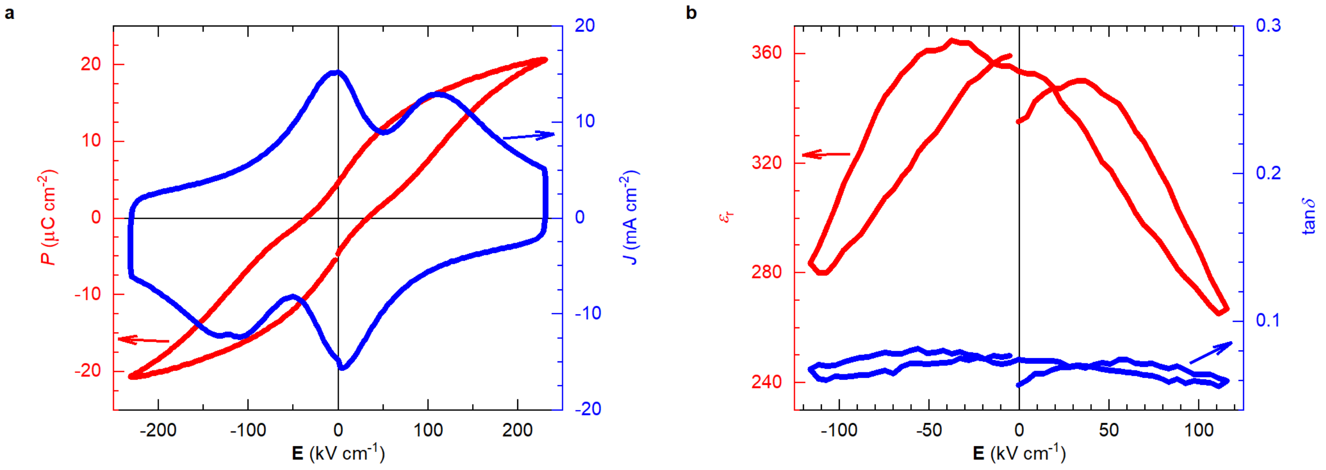

Supplementary Figure 12. **Ferroelectric and dielectric characterizations** of 1 µm thick PZT actuator on Schott AF32 glass. a) $P(\mathbf{E})$ and $J(\mathbf{E})$ loops of per actuator, measured at 100 Hz. b) corresponding $\varepsilon_r(\mathbf{E})$ and $\tan\delta(\mathbf{E})$ loops of the actuator, measured as functions of DC voltage with a probing AC signal of 0.5 V at 1 kHz.

Supplementary Note 4: Flash lamp process for growing films on soda lime glass

Supplementary Note 4.1: Two-step flash lamp annealing process

The one-step process used for fused silica and AF32 glass is not suitable for growing PZT film on soda lime glass due to appearance of cracks. This can be attributed to the low thermal conductivity (1.0 W m^{-1} K^{-1}) of the substrate, which leads to a slower rate of heat transfer and consequently a higher temperature at the interface between the film and glass.

To address this issue, we have developed a two-step process consisting of stages where either nucleation or growth is dominating. In the first step, pulses with higher power density are applied to induce the formation of nuclei within the film. This formation of nuclei reduces the activation energy required for the phase transition from an amorphous to a crystalline phase. In the second step, the phase where crystal growth is dominating, pulses with a lower power density are applied to grow the film at a lower temperature, thereby preventing the occurrence of cracks.

Supplementary Note 4.2: Phase composition characterization

Supplementary Figure 13 displays GIXRD patterns of PZT films with thicknesses of 170 and 500 nm deposited on soda lime glass. The dominant reflections in both patterns correspond to the piezoelectrically active perovskite phase, suggesting that the FLA process is suitable for layer-by-layer preparation in solution processing. This is particularly significant for various applications. Although a weak reflection at approximately 29° indicates the presence of the secondary pyrochlore phase, its relative intensity is considerably lower than that of the perovskite reflections.

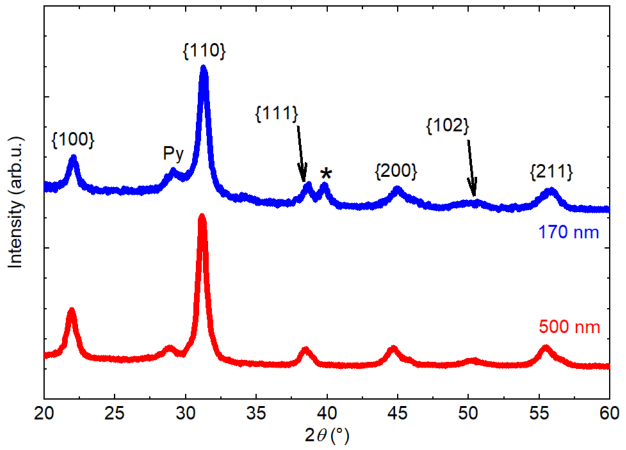

Supplementary Figure 13. **XRD study**. GIXRD patterns of 170 nm- and 500 nm-thick PZT films on soda lime glass. * denotes the signal of Pt IDEs on top of the PZT film. The PDFs No 01-070-4264 and 04-014-5162[3] have been used to identify the perovskite and pyrochlore phases, respectively.

Supplementary Note 4.3: Electrical measurements

The P(**E**) and J(**E**) loops of a 170 nm-thick PZT thin film on soda lime are shown in Supplementary Figure 14 along with its dielectric characterization.

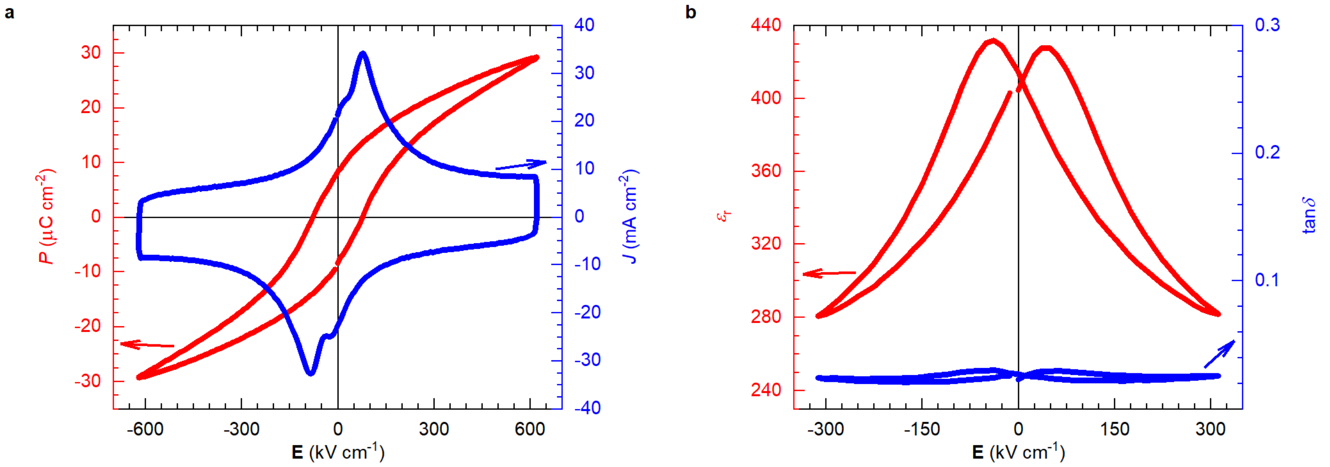

Supplementary Figure 14. **Ferroelectric and dielectric characterizations**. Ferroelectric and dielectric measurements of 170 nm-thin PZT on soda lime glass. a) P(**E**) and J(**E**) loops, measured at 100 Hz. b) ε_r(**E**) and tanδ(**E**) loops, measured at 1kHz. Small IDEs was used, whose parameters are 5 μm in digit width, 3 μm in interdigital gap, 50 pairs of digits and 370 μm in length of digits facing each other.

Supplementary Note 5: Comparison with previous works

Supplementary Table 3: **Summary** of relevant points in the references previously reporting FLA treatment of PZT films and major advancement shown in this work.

**Reference**	**Relevant points in the reference**	**Major advancement in our work compared to reference**
Yamakawa et al., Jpn. J. Appl. Phys., 41 2630 (2002)[13].	• FLA treatment of PZT thin films.<br>• Ambient temperature between 300 and 500 °C.<br>• Crystalline perovskite phase present prior FLA treatment.<br>• No piezoelectric properties.	• Ambient environment at room temperature.<br>• Crystallization of completely amorphous initial films.<br>• Demonstration of piezoelectric properties and a device.
Yao et al., J. Eur. Ceram. Soc., 40, 5396 (2020)[14].	• FLA treatment of PZT thin films.<br>• No macroscopic electromechanical characterization (films too leaky).	• Demonstration of macroscopic electromechanical properties and a device.
Palneedi et al., Adv. Mater., 2303553 (2023)[15].	• FLA sintering of crystalline PZT powders deposited on metglas (amorphous metal).	• In-situ FLA crystallization of amorphous PZT thin films.
Ouyang et al., J. Am. Ceram. Soc., 99, 2569 (2016)[16].	• FLA sintering of crystalline PZT powders on stainless steel.<br>• Non-saturated P-E loops.	• In-situ FLA crystallization of amorphous PZT thin films.<br>• Good ferroelectric properties.
Ouyang, PhD Thesis, Rochester Institute of Technology (2017)[17].	• FLA sintering crystalline PZT powders on stainless steel and PET substrates.<br>• Non-saturated P-E loops.	• In-situ FLA crystallization of amorphous PZT thin films.<br>• Good ferroelectric properties.
Marotta, MSc Thesis, Rochester Institute of Technology (2019)[18].	• FLA treatment of printed PZT thin films.<br>• No crystallization (XRD) reported.<br>• No macroscopic electromechanical characterization.	• In-situ FLA crystallization of amorphous PZT thin films.<br>• Demonstration of macroscopic electromechanical properties.

Two major points are stemming from the Table:

1) In all the previous reports with demonstrated macroscopic functional properties, FLA sintering was performed on already crystalline PZT powders. In this work, FLA crystallization, i.e., nucleation of perovskite grains and their growth, was performed from amorphous films (see Figure 2a of the main article). Perovskite formation is nucleation-

controlled, with activations energies for nucleation and grain growth of 441 kJ mol^{-1} and 112 kJ mol^{-1}, respectively[19]. In other words, the most energetically demanding process for perovskite formation is nucleation from the amorphous phase, which this study is the only one to demonstrate with FLA.

2) In the remaining reports, where they worked on FLA treatment of amorphous PZT films, macroscopic ferroelectric results could not be obtained. The only exception is the work of Yamakawa et al.[13] on sputtered PZT films. In that case, the FLA treatment was performed at elevated ambient temperatures and XRD reveals the presence of the perovskite phase already before FLA treatment (Figure 8 in the article). Therefore, this work is the only one that shows macroscopic ferroelectric results starting from fully amorphous films.

Supplementary Table 4: **FLA processing parameters taken from the literature and compared to the parameters used in this work.** Note that Ouyang's PhD thesis[17] is omitted as its results are summarized in the article in Journal of the American Ceramics Society[16]. Marotta's MSc thesis[18] is omitted from this analysis also, as little information on FLA process is given. *Energy per pulse in Yamakawa's work[13] is estimated from the current delivered to the Xe lamp when the voltage is applied. Real energy delivered to the sample is probably much lower. $^{+}$Power per pulse (unless given) is estimated from the energy divided by pulse width. $^{\#}$In this work energy delivered to the sample was measured using bolometer.

**Reference**	**Energy per pulse (J cm^{-2})**	**Pulse width (µs)**	**Power per pulse^{+} (kW cm^{-2})**	**Number of pulses**	**Pulse frequency (Hz)**
Yamakawa et al., Jpn. J. Appl. Phys., 41 2630 (2002)[13].	27*	1000-1500	18-27	up to 5	not given
Yao et al., J. Eur. Ceram. Soc., 40, 5396 (2020)[14].	not given	250-500	Up to 6.4	up to 100	not given
Palneedi et al., Adv. Mater., 2303553 (2023)[15].	1.7 – 7.4	250-1000	7	up to 3	1
Ouyang et al., J. Am. Ceram. Soc., 99, 2569 (2016)[16].	2.8	1300	2.2	Up to 15	2
This work	3$^{\#}$	130	up to 23	Up to 100	3

Supplementary References

1. Guillot, M. J., McCool, S. C. & Schroder, K. A. Simulating the thermal response of thin films during photonic curing. *ASME Int. Mech. Eng. Congr. Expo. Proc.* **7**, 19–27 (2013).

2. Piper, R. T., Daunis, T. B., Xu, W., Schroder, K. A. & Hsu, J. W. P. Photonic Curing of Nickel Oxide Transport Layer and Perovskite Active Layer for Flexible Perovskite Solar Cells: A Path Towards High-Throughput Manufacturing. *Front. Energy Res.* **9**, 1–12 (2021).

3. ICDD database PDF4+ v.19. (2019).

4. Glinsek, S. *et al.* Fully transparent friction-modulation haptic device based on piezoelectric thin Film. *Adv. Funct. Mater.* **30**, 2003539 (2020).

5. Nguyen, C. H. *et al.* Probing-models for interdigitated electrode systems with ferroelectric thin films. *J. Phys. D. Appl. Phys.* **51**, 175303 (2018).

6. Muralt, P. Recent progress in materials issues for piezoelectric MEMS. *J. Am. Ceram. Soc.* **91**, 1385–1396 (2008).

7. AF32 glass datasheet. https://www.pgo-online.com/intl/af32.html (2022).

8. Song, L. *et al.* Piezoelectric thick film for power-efficient haptic actuator. *Appl. Phys. Lett.* **121**, 212901 (2022).

9. Bernard, F., Casset, F., Danel, J. S., Chappaz, C. & Basrour, S. Characterization of a smartphone size haptic rendering system based on thin-film AlN actuators on glass substrates. *J. Micromechanics Microengineering* **26**, 84007 (2016).

10. Bernard, F. Conception, fabrication et caractérisation d'une dalle haptique à base de microactionneurs piézoélectriques. (Université Grenoble Alpes, 2016).

11. Hua, H., Chen, Y., Tao, Y., Qi, D. & Li, Y. A highly transparent haptic device with an extremely low driving voltage based on piezoelectric PZT films on glass. *Sensors Actuators A Phys.* **335**, 113396 (2022).

12. Glinsek, S. *et al.* Inkjet-printed piezoelectric thin films for transparent haptics. *Adv. Mater. Technol.* **7**, 2200147 (2022).

13. Yamakawa, K. *et al.* Novel Pb(Ti, Zr)O3 (PZT) crystallization technique using flash lamp for ferroelectric RAM (FeRAM) embedded LSIs and one transistor type FeRAM devices. *Japanese J. Appl. Physics, Part 1 Regul. Pap. Short Notes Rev. Pap.* **41**, 2630–2634 (2002).

14. Yao, Y. *et al.* Direct processing of PbZr0.53Ti0.47O3 films on glass and polymeric substrates. *J. Eur. Ceram. Soc.* **40**, 5369–5375 (2020).

15. Palneedi, H. *et al.* Intense pulsed light thermal treatment of Pb(Zr,Ti)O3/metglas heterostructured films resulting in extreme magnetoelectric coupling of over 20 V cm-1 O-1. *Adv. Mater.* 2303553 (2023) doi:10.1002/adma.202303553.

16. Ouyang, J., Cormier, D., Williams, S. A. & Borkholder, D. A. Photonic sintering of aerosol jet printed lead zirconate titanate (PZT) thick films. *J. Am. Ceram. Soc.* **99**, 2569–2577 (2016).

17. Ouyang, J. Enhanced piezoelectric performance of printed PZT films on low temperature substrates. (Rochester Institute of Technology, 2017).

18. Marotta, A. R. Printable thin-film sol-gel lead zirconate titanate (PZT) deposition using nanojet and inkjet printing methods. (Rochester Institute of Technology, 2019).

19. Chen, K. C. & Mackenzie, J. D. Crystallization kinetics of metallo-organics derived PZT thin film. *MRS Online Proc. Libr.* **180**, 663–668 (1990).
